# Two-dimensional graphene–HfS_2_ van der Waals heterostructure as electrode material for alkali-ion batteries†

**DOI:** 10.1039/d0ra04725b

**Published:** 2020-08-17

**Authors:** Gladys W. King'ori, Cecil N. M. Ouma, Abhishek K. Mishra, George O. Amolo, Nicholas W. Makau

**Affiliations:** University of Eldoret P.O. Box 1125 – 30100 Eldoret Kenya gking.kingori@gmail.com; Technical University of Kenya Haile Selassie Avenue, P.O. Box 52428 – 00200 Nairobi Kenya; HySA-Infrastructure, North-West University, Faculty of Engineering Private Bag X6001 Potchefstroom 2520 South Africa; Department of Physics, School of Engineering, University of Petroleum and Energy Studies Bidholi via Premnagar Dehradun 248007 India

## Abstract

Poor electrical conductivity and large volume expansion during repeated charge and discharge is what has characterized many battery electrode materials in current use. This has led to 2D materials, specifically multi-layered 2D systems, being considered as alternatives. Among these 2D multi-layered systems are the graphene-based van der Waals heterostructures with transition metal di-chalcogenides (TMDCs) as one of the layers. Thus in this study, the graphene–hafnium disulphide (Gr–HfS_2_) system, has been investigated as a prototype Gr–TMDC system for application as a battery electrode. Density functional theory calculations indicate that Gr–HfS_2_ van der Waals heterostructure formation is energetically favoured. In order to probe its battery electrode application capability, Li, Na and K intercalants were introduced between the layers of the heterostructure. Li and K were found to be good intercalants as they had low diffusion barriers as well as a positive open circuit voltage. A comparison of bilayer graphene and bilayer HfS_2_ indicates that Gr–HfS_2_ is a favourable battery electrode system.

## Abstract 2

A high rate capacity, moderate volume expansion and energetically stable alkali ion graphene–HfS_2_ electrode material.

## Graphical abstract

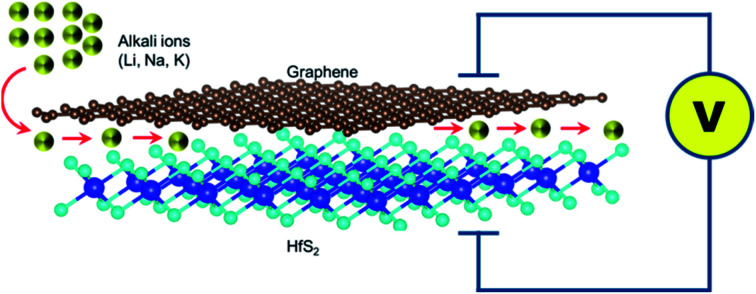

## 1 Introduction

Rechargeable battery electrode materials suffer from poor electrical conductivity and large volume expansion during repeated charge and discharge, which neutralizes their large capacity and impairs their long term electrochemical stability.^1^ This has led to studies on how electrode materials can be modified either *via* doping or creation of Gr based two-dimensional (2D) van der Waals heterostructures, notably those based on transition metal di-chalcogenides (TMDCs). 2D van der Waals heterostructures afford an opportunity to develop rechargeable battery storage systems with high rate capacity and storage density as well as cyclic stability.^2,3^ Due to the challenges facing electrode materials such as low gravimetric and volumetric energy densities, there is need for materials with possible higher gravimetric and volumetric energy densities. However, many of them suffer from limited electrical conductivity, slow lithium transport, large volume expansion, low thermal stability, mechanical brittleness, and dissolution as well as other unsuitable interactions with the battery electrolyte.^4^

2D materials offer several favorable properties over their 3D counterparts especially in the design of next generation devices.^5,6^ Graphene a pioneer 2D material has been widely investigated due to it being very thin, highly transparent, very flexible, having large surface area, outstanding conductivity^7^ and good stability for chemical agents.^8^ These properties make it suitable for transparent conducting electrodes applications^7^ as well as for energy storage.^9^ However, despite its attractive properties, the lack of finite gap has been its main caveat in nanoelectronic applications.^10,11^ It also exhibits severe aggregation and restacking which results in a much lower specific surface area. Low specific surface area leads to ions not accessing the surface of the electrode, and this affects an electrodes' cyclic ability.^12^ Additionally, Gr has low storage capacity for alkali ions.^13,14^

Two-dimensional transition metal dichalcogenides (2D TMDCs) on the other hand, are a family of materials whose generalized formula is MX_2_, where M represents transition metal and X represents the chalcogenide elements.^15^ These materials are almost as thin, transparent and flexible as graphene, however unlike graphene, TMDCs have a diversity of chemical compositions and structural phases that results in a broad range of electronic properties, both from the point of view of the emergence of correlated and topological phases and of the band structure character (metallic or insulating).^16,17^ Existence of semiconductor TMDCs means that they have the prospects for a wide range of applications.^18–21^ HfS_2_ is one such TMDC with an indirect energy band gap of ∼1.30 eV ^22^ a good upper limit of mobility (∼1800 cm^2^ V^−1^ s^−1^),^23^ and bonds that are more ionic than those in MoS_2_.^24^ As a result, the charge transfer per S atom in HfS_2_ is expected to be higher.^24^

Monolayer TMDCs often exist in two basic phases; the trigonal prismatic referred to as the 1H and the octahedral phase referred to as the 1T phase. In the 1T phase there is the undistorted 1T phase, where the metal atom is located at the centre of an octahedral unit and distorted 1T phase (called the 1T′ phase), in which pairs of metal atoms move closer to each other perpendicularly, resulting in a quasi-one-dimensional chain-like structure consisting of distorted octahedral units as well as another distorted 1T phase (called the 1T′′ phase), in which four nearby metal atoms move closer to each other to form a new unit cell, producing repeatable diamond-like pattern.^25^ However, HfS_2_ is known to crystallize in the 1T type structure, since its other phases are unstable,^26,27^ thus, in this study 1T phase of HfS_2_ is considered.

Another important property of TMDCs is that they possess weak van der Waals interaction between the respective TMDCs layers, this makes it possible to stack different TMDCs layers to form heterostructures with new electronic properties. Graphene based heterostructures have been created by using graphene as one of the layers forming the heterostructure. This has already been done in the case of Gr/MoS_2_,^28^ Gr/WS_2_ ^29^ and Gr/VS_2_.^30^

Studies have also reported the possibility of alkali ions intercalation in these van der Waals heterostructures with binding energies per intercalated ion as well as band gap increasing with increase in the number of intercalated ions.^31,32^ Alkali ion intercalation has been found to lead to the vertex of the Dirac cone shifting downward due to n-doping of the Gr monolayer by the electrons transferred from intercalated atoms.^30^ In addition, such heterostructures have the potential to overcome the restacking problem of pure Gr.^33^

In this study, using dispersion corrected density functional theory (vdW-DFT), alkali ion intercalation in Gr–HfS_2_ van der Waals heterostructure has been investigated to determine the interlayer binding energy, identify the minimum energy configuration of the Gr–HfS_2_ heterostructure as well as investigate the influence of intercalants (Li, Na and K) on the properties of the Gr–HfS_2_ heterostructure, among others.

## 2 Computational details

In this work, first-principles calculations were performed within the density functional theory (DFT) framework, as implemented in Quantum ESPRESSO code.^34^ The study used the Perdew–Burke–Ernzerhof (PBE) functional^35^ to describe the electrons exchange–correlation potentials. Interlayer van der Waals (vdW) interactions of the Gr–HfS_2_ systems were considered in all the calculations through the van der Waals density functional (vdW-DF2) scheme.^36^ To include the electron–ion interaction, norm-conserving pseudopotentials^37^ were used for all the atoms. Monolayers of Gr and HfS_2_ were obtained from their bulk counterparts whose equilibrium properties were obtained using a converged kinetic energy cut-off of 70 Ry, Gamma-centred *k*-point mesh of 8 × 8 × 3 for graphite and 7 × 7 × 4 for HfS_2_. A convergence criteria of 10^−6^ Ry in calculated total energies was imposed on all the systems investigated. Optimized lattice constants were obtained using the PBE functional with and without the vdW-DF2, for the purpose of illustrating the role of the van der Waals (vdW) interactions in these layered materials.

**Fig. 1:**
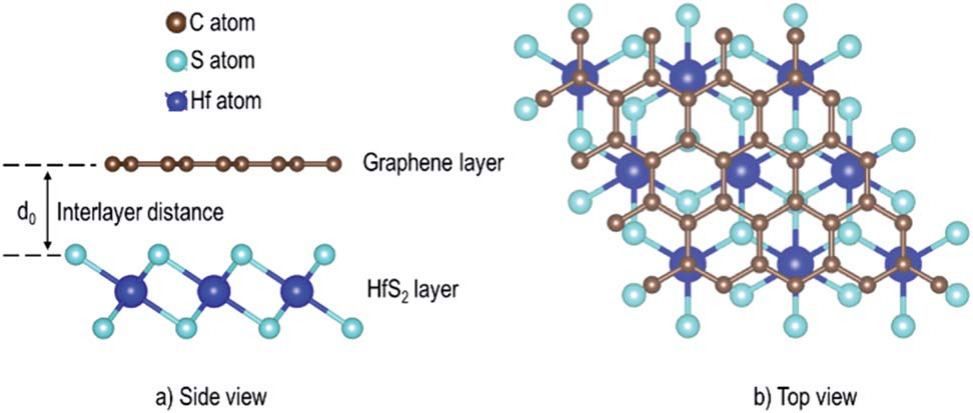
Schematic illustration of the Gr–HfS_2_ heterostructure with the most energetically stable configuration.

Monolayer unit cells of Gr and HfS_2_ were then created from the bulk systems and a 15 Å vacuum was added along the direction perpendicular to the atomic planes of the bulk structures of graphite and HfS_2_, respectively. The vacuum helps to minimize the interaction between the layers along the *c*-axis. The atomic positions of the monolayer systems were relaxed keeping the volume fixed. The heterostructure was then constructed by placing the Gr monolayer on top of the HfS_2_ monolayer. However, due to the difference in the equilibrium lattice constants of Gr and HfS_2_, there was need to reduce the lattice mismatch in the created heterostructure. This was done by creating supercells of different sizes for each of the monolayers. Supercell sizes of 3 × 3 × 1 and 2 × 2 × 1 for Gr and HfS_2_, respectively, were used in creating the heterostructure (see Fig. 1) as this is what resulted in a small lattice mismatch between the Gr and HfS_2_ layers. The lattice mismatch was obtained as (eqn (1)),1
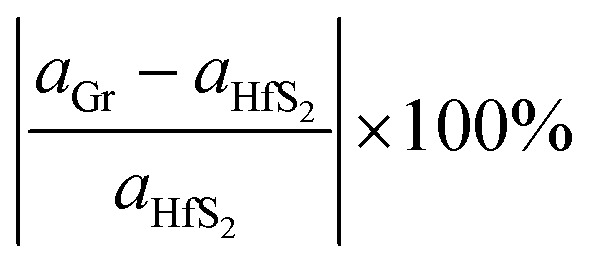
where *a*_Gr_ and *a*_HfS2_ is the lattice constants of Gr and HfS_2_ supercells respectively.

First-principles calculations with the climbing image nudged elastic band (CI-NEB)^38^ method, as implemented in the Quantum ESPRESSO transition state tools was employed to investigate the energy barrier associated with the migration of the Li, Na and K atoms through the heterostructure. For comparison, diffusion through bilayer Gr and bilayer HfS_2_ was also considered.

## 3 Results and discussion

The optimized lattice constants of the bulk structures of graphite and HfS_2_ calculated using the PBE functional with and without the vdW-DF2 are presented in Table 1. It is observed from Table 1, that the PBE functional describes the covalent bonds inside the graphene and HfS_2_ fairly well, and this results in very good agreement between the values of the lattice constant ‘*a*’ obtained using PBE functional with and without the vdW-DF2, as well as with experimental data. It is observed from Table 1, that the calculated interlayer distances from PBE functional with vdW-DF2 scheme, for both graphite and bilayer HfS_2_ are in good agreement with experimentally observed interlayer distances. However, in comparison to the experimental values, PBE without vdW-DF2 significantly overestimates the interlayer distance by 0.7 Å (20.8%) and 1.08 Å (18.6%) for graphite and HfS_2_, respectively. This can be attributed to the fact that the covalent interactions are dominant along the plane of the structures in comparison to the weak van der Waals interactions between the layers. Therefore, inclusion or omission of vdW-DF2 scheme when determining lattice parameter ‘*a*’ has minimal effects, however ignoring it when determining interlayer distances, results in unreliable results.

**Table 1:** Calculated lattice constants ‘*a*’ and interlayer distance *d*_0_ with and without vdW-DF2

		*a* (Å)	*d*_0_ (Å)
Graphite	PBE	2.47	3.43
	vdW-DF2	2.46	3.35
	From experiment	2.46 ^39^	3.36 ^39,40^
HfS_2_	PBE	3.66	6.93
	vdW-DF2	3.64	5.82
	From experiment	3.63 ^41^	5.85 ^41^

These observations indicate that the PBE functional with vdW-DF2 scheme accounts for the weak interlayer vdW interactions, and hence was adopted for the study.

The binding energy, *E*_b_ was obtained as.2
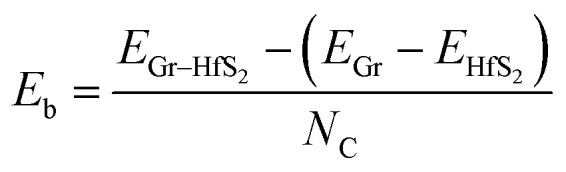
where, *E*_Gr–HfS2_, *E*_Gr_ and *E*_HfS2_ are the calculated total energies of the Gr–HfS_2_ heterostructure, Gr monolayer and HfS_2_ monolayer, respectively, and *N*_C_ is the total number of C atoms in the system. By this definition (eqn (2)), the configuration with the lowest binding energy is the low energy configuration adopted for subsequent investigation. As seen in Table 2, the configuration with Gr lattice constant as reference was the one associated with lowest binding energy hence the configuration of choice even though it was not the one with lowest lattice mismatch. This configuration had a lattice mismatch of 1.37%. Other studies on heterostructures have reported lattice mismatches of 1.7% for graphene/Ti_2_CO_2_ ^42^ and graphene/*h*BN^43,44^ and 2.37% in HfS_2_/MoTe_2_.^45^

**Table 2:** Binding energies corresponding to various Gr–HfS_2_ heterostructure configurations. *E*_b_ is the binding energy per carbon

	*E*_b_	Lattice mismatch
Gr as the reference	−0.040 eV	1.37%
HfS_2_ as a reference	0.038 eV	1.35%
Gr and HfS_2_ as reference	−0.017 eV	0.70%

**Fig. 2:**
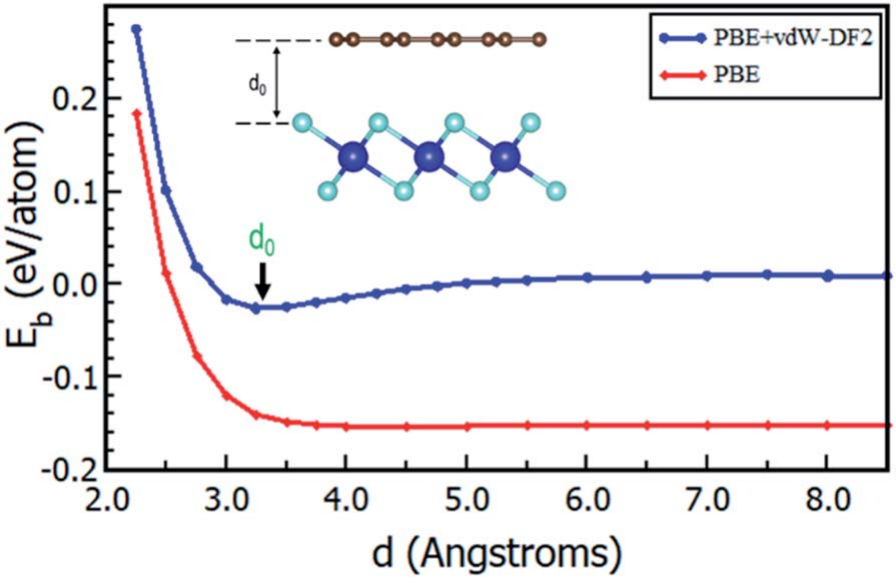
Binding energy of Gr–HfS_2_ van der Waals heterostructure with and without vdW-DF2 as a function of the interlayer distance, *d*. Image inset shows the interlayer distance, *d*.

Having chosen the configuration with Gr lattice constant as reference, we endeavoured to determine the equilibrium interlayer distance of this configuration. Using eqn (2), where *E*_b_ was calculated at different interlayer distances, *d*, it can also be argued that, a lower *E*_b_ value means a more stable heterostructure and *vice versa*. The calculated value of binding energies per C atom with and without van der Waals corrections at different interlayer distances are presented in Fig. 2. It is evident that the inclusion of van der Waals corrections resulted in a Lennard Jones potential-type^46^ with a distinct equilibrium interlayer distance. This observation was however absent in the case when van der Waals corrections were not included in the calculations, hence subsequent calculations only considered instances where van der Waals corrections were included. The plot of PBE with van der Waals corrections implies that, when the layers are bought very close, repulsive forces come into play. However, when the layers are pulled further apart, the attractive forces intended to draw the layers closer together are negligible, and hence the importance of including the vdW interactions when considering the Gr–HfS_2_ heterostructure.

The calculated equilibrium interlayer distance *d*_0_ was 3.30 Å and the corresponding binding energy was −140 meV. Other studies have established that for bilayer graphene, the interlayer binding energy is −11.5 meV ^47^ and −10.4 meV.^48^ In other analogous systems, *d*_0_ was found to be 3.33 Å for bilayer Gr,^49^ 3.1 Å for MoS_2_/Gr systems,^49^ 3.22 Å for hexagonal-Boron Nitride/Gr (h-BN/Gr) hetero-bilayer,^50^ 3.25 Å for graphene/Pt_2_HgSe_3_ heterostructure,^51^ and 3.75 Å for graphene/graphene-like germanium carbide heterostructure.^52^ On the other hand, *E*_b_ has been reported as −78 meV for graphene/Pt_2_HgSe_3_ heterostructure,^51^ −28 meV for graphene/h-BN heterostructure,^53^ −38 meV for graphene/graphene-like germanium carbide heterostructure^52^ and 51 meV for Gr/MoS_2_ heterostructure.^49^ The negative binding energies of the Gr–HfS_2_ heterostructure confirm the thermodynamic stability of the heterostructure and the increased binding energy in comparison to that of bilayer Gr could also help overcome the restacking problem common in graphene. All subsequent calculations, were done using the obtained *d*_0_.

### 3.1 Electronic properties

**Fig. 3:**
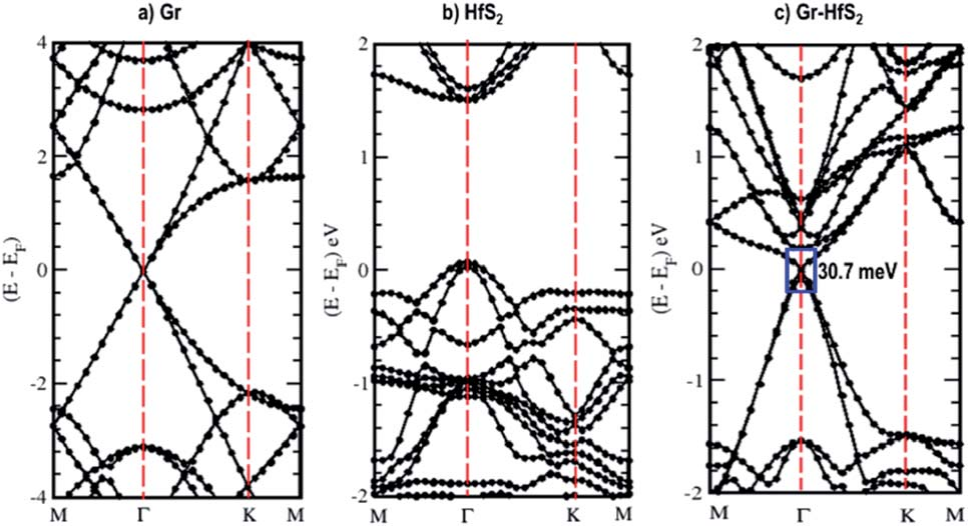
Calculated electronic band structures of 3 × 3 × 1 Gr supercell, 2 × 2 × 1 HfS_2_ supercell and Gr–HfS_2_ heterostructure system.

**Fig. 4:**
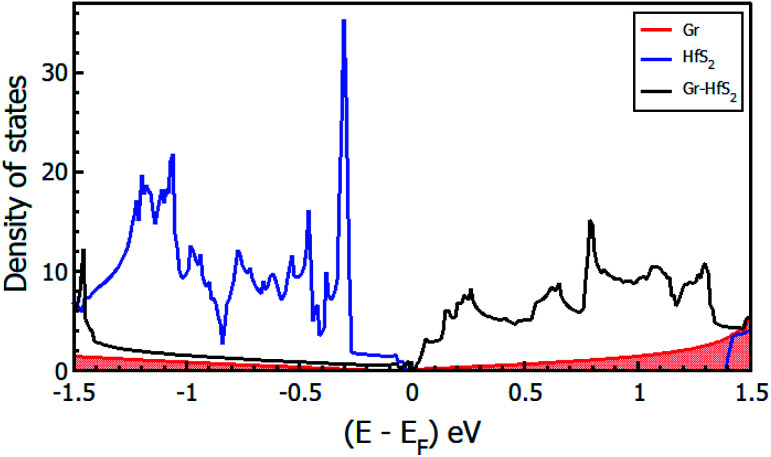
Density of states of the Gr–HfS_2_ heterostructure system projected on the 3 × 3 × 1 Gr supercell and 2 × 2 × 1 HfS_2_ supercell.

**Fig. 5:**
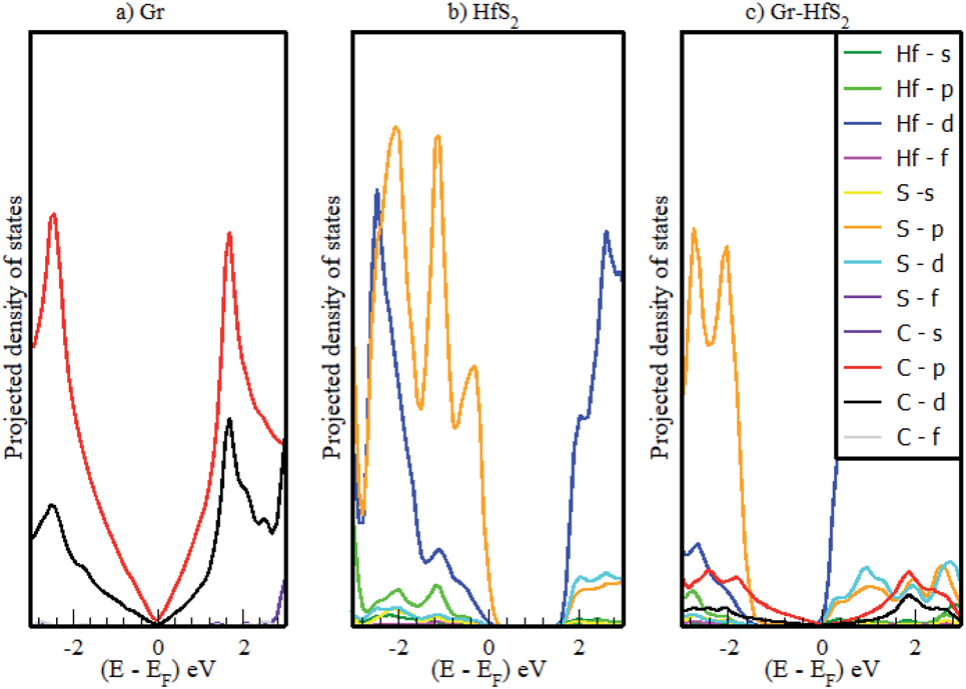
Calculated projected density of states of (a) 3 × 3 × 1 Gr supercell, (b) 2 × 2 × 1 HfS_2_ supercell and (c) Gr–HfS_2_ heterostructure system.

The calculated band structures and their respective DOS and PDOS for Gr, HfS_2_ and Gr–HfS_2_ heterostructure are shown in Fig. 3–5. Gr is semi metallic while HfS_2_ is a semi conductor having a band gap of 1.30 eV.^22^ The monolayer of HfS_2_ (Fig. 3b), was found to have a direct electronic band gap of 1.45 eV, which compares well with previous studies that found the band gap to be 1.28 eV ^54^ and 1.30 eV.^22^ As can be seen in Fig. 3, the weak interaction between the two layers in the Gr–HfS_2_ vdW heterostructure resulted in a vanishingly small bandgap (30.7 meV) opening at gamma point. This observation is also consistent with previous graphene based heterostructures where electronic band gaps of the same order were observed. As examples, Pelotenia *et al.*^50^ observed an electronic band gap in hexagonal Boron Nitride/Gr hetero-bilayer of 20 meV, while Yuan *et al.*^55^ found a band gap of 11 meV for Gr/WS_2_. Other studies have also found equally small band gaps such as 0.4 meV for Gr/MoS_2_.^56^ As can be seen in Fig. 4, the band gap opens within the region where the electronic gap for HfS_2_ is found. By further considering the PDOS for the heterostructure (see Fig. 5), it is observed that the electronic band gap originates from an interaction mainly between the C-p and Hf-d orbitals. It is equally important to note that a desirable electrode material ought to be a good conductor in order to facilitate the movement of electrons thus, it should possess a negligible band gap. The calculated band structures (Fig. 3) indicate that the Gr–HfS_2_ heterostructure has a band gap of 30.7 meV hence can function as a good electrode. This implies that an electron in the valence band of the heterostructure require very little energy (30 meV) to move to the conduction band. Electronic conductivity plays a significant role during the (de)intercalation of charge-carrying ions within an electrode material, since it influences the efficient movement of electrons and ions especially at high current rates.^57^ In cases when fast acceleration is needed as in electric vehicles, alkali ions and/or electrons should be able to move through the material quickly enough to utilize all stored chemical energy. The negligible electronic band gap of the Gr–HfS_2_ heterostructure would therefore be expected to lead to efficient movement of electrons in the electrode.

Mapping the Gr–HfS_2_ heterostructure DOS onto that of Gr and HfS_2_ monolayers (Fig. 4), indicate that the valence band of heterostructure is dominated by the Gr layer while the conduction band is dominated by the HfS_2_ layer. PDOS plots (Fig. 5), indicate that the p orbital of C in Gr dominate the edges of the Dirac cone in graphene's band structure while, the d orbital of Hf formed the conduction band edge of both HfS_2_ monolayer as well as Gr–HfS_2_ heterostructure.

### 3.2 Work-function of the heterostructure

**Fig. 6:**
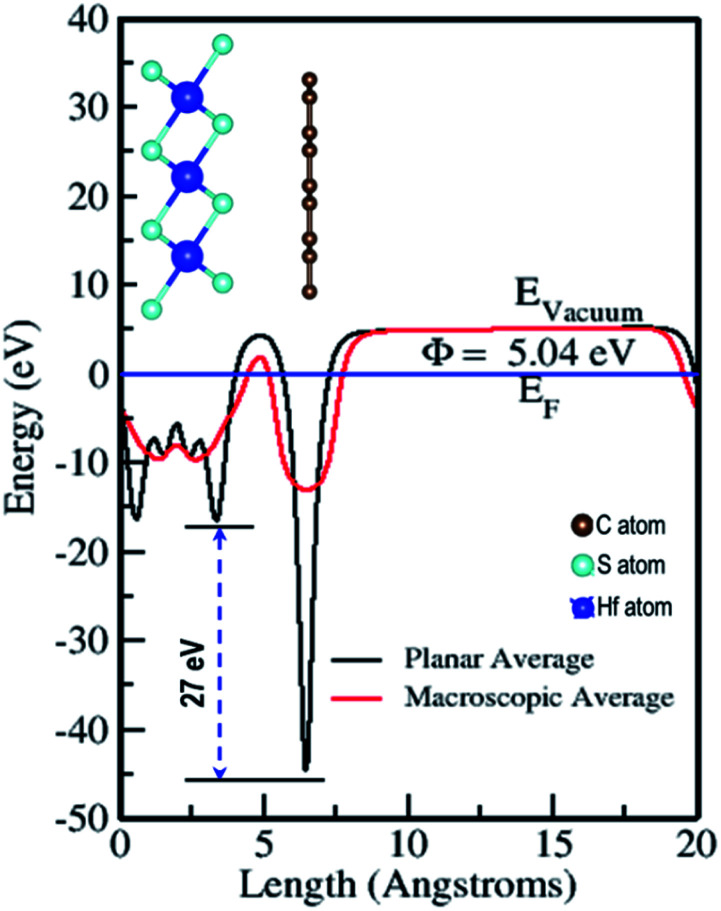
Planar and macroscopic electrostatic potentials for Gr–HfS_2_ heterostructure.

The electrostatic potential of the Gr–HfS_2_ heterostructure was obtained along the *z*-direction (Fig. 6), where the vacuum level is the region outside the surface where the potential reaches a constant (flat level). The vacuum level was determined from the calculated macroscopic and planar averages of the electrostatic potential. Using this, the work function was then calculated using the equation,

3 *Φ* = *E*_vac_ − *E*_F_

where *E*_vac_ is the electrostatic potential in the vacuum region while *E*_F_ refers to the Fermi energy.^58^ The calculated values of the work function for Gr and HfS_2_ were 4.25 eV and 6.20 eV, respectively, both of which were equal to previous studies.^59^ The calculated work function for the Gr–HfS_2_ heterostructure was 5.04 eV, implying that Gr decreases the work function of HfS_2_ upon formation of the heterostructure, this in turn, makes it easier for electrons to be lost to the surface. The planar average potential around Gr consisted of a single distinct hump that corresponded to the monolayer of Gr, while the part around the HfS_2_ consisted of three (3) peaks corresponding to the three sublayers of S, Hf and S, respectively. The electrostatic potential of Gr is deeper compared to that of HfS_2_, and this results in a large potential drop of 27 eV across the *z*-direction of the heterostructure. This can be attributed to the differences in the atomic electronegativity of S = 2.58, Hf = 1.3 and C = 2.5.^60^ Hence, it is expected that electrons will be transferred from the Gr layer to the HfS_2_ layer.^61^ The large potential drop of 27 eV suggests a powerful electrostatic field across the interface, so that when the Gr layer is used as an electrode, this field will considerably affect the carrier dynamics and induce a low charge-injection barrier which will facilitate charge injection.^62^

### 3.3 Alkali ion intercalation

Intercalation is the reversible insertion of foreign species into the gap/space of a crystal or layers. Layered materials are good host materials for various intercalant species ranging from small ions, to atoms and even to molecules.^63^ Layered crystals are particularly suitable for intercalation as they can strongly adsorb guest species in their van der Waals interlayer spacing(s).^63^ In this study, the alkali ion(s) were inserted between the two layers of the Gr–HfS_2_ heterostructure. A systematic study of intercalating different alkali ion species namely Li, Na and K in the Gr–HfS_2_ heterostructure was carried out. This was informed by the fact that alkali ions such as Li have low reduction potentials that make their intercalation in battery materials attractive. Li is also the third lightest element with one of the smallest ionic radius of 2.20 Å.^64^ The ionic radii of the other two alkali atoms, Na and K, are 2.25 Å and 2.34 Å, respectively.^64^ It was anticipated that these other alkali ions, that is Na and K, might have similar properties as Li and hence the reason for their inclusion in this study. In addition and more importantly they are considerably more accessible than lithium.^65^ The most energetically favorable position for the intercalants (with Li used as a test case) was established through the calculation of the binding energy with the intercalant in different positions. The binding energy in these systems was calculated as (eqn (4)),4
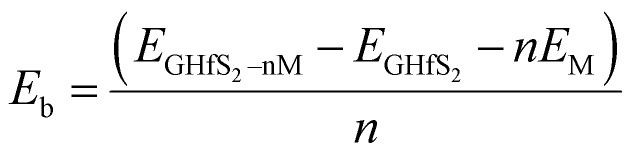
where *E*_GHfS2–nM_ is the total energy of the Gr–HfS_2_ heterostructure with the alkali adatom, *E*_GHfS2_ is the total energy of the Gr–HfS_2_ heterostructure without any alkali adatom, *E*_M_ is the total energy of the free metal adatom, and *n* corresponds to the number of alkali ions.

**Fig. 7:**
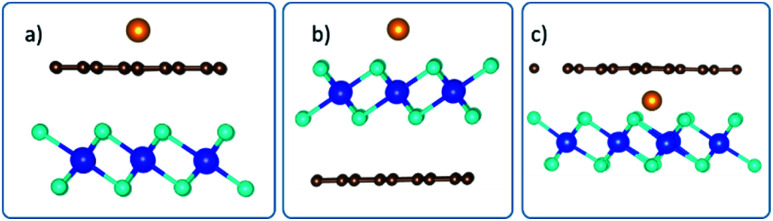
Side views of adsorption of intercalant, Li, on the Gr–HfS_2_ vdW heterostructure with intercalant (a) above Gr (b) above HfS_2_ and (c) in between Gr and HfS_2_ layers respectively.

Using the configuration presented in Fig. 7, the binding energies for the system when intercalant is adsorbed on top of Gr, above HfS_2_ and in between the Gr and HfS_2_ layers of the Gr–HfS_2_ heterostructure were 0.4 eV, −1.0 eV and −1.6 eV, respectively, indicating that the system with Li between the layers is most stable.

**Fig. 8:**
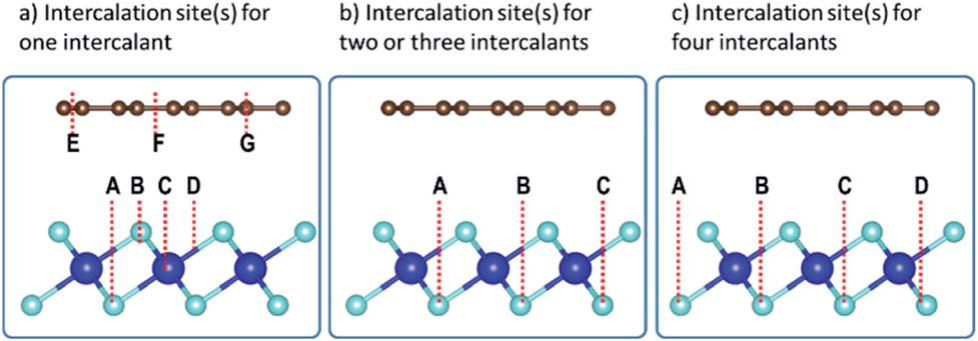
Intercalation sites for (a) one, (b) two/three and (c) four intercalants, respectively.

The preferred intercalation site(s) was(were) then identified by inserting the intercalant at different sites between the Gr and HfS_2_ layers. There are several sites accessible to the intercalant between the Gr and HfS_2_ layers (Fig. 8a). In determining the lowest energy intercalant site, the binding energy was calculated with the intercalant atoms at positions A, B, C, D, E, F and G as shown in Fig. 8a.

As seen in Table 3, intercalant site A was favored as it was the one with the lowest binding energy. Three other identical sites to A were determined by symmetry and were then considered as sites for adding two, three and four intercalants (Fig. 8b and c) between the layers of the heterostructure. As seen in Fig. 8b there are two distinct configurations we identified (intercalants at sites A and B and intercalant at sites A and C) that could be used to intercalate two intercalant atoms between the layers. The binding energy associated with positions A and B was −0.172 eV while that for positions A and C was −0.171 eV. As a result, intercalation of two atoms was done using a configuration similar to that of positions A and B. Only one configuration was possible for the three and four intercalant atoms intercalation (see Fig. 8b and c). The number of intercalated ions was therefore sequentially increased from 1 to 4.

**Table 3:** Binding energies of intercalant atoms at sites indicated in Fig. 8a

Position	*E*_b_ (eV)
A	−0.091
B	−0.089
C	−0.088
D	−0.079
E	−0.090
F	−0.088
G	−0.089

### 3.4 Effect of intercalant concentration

**Fig. 9:**
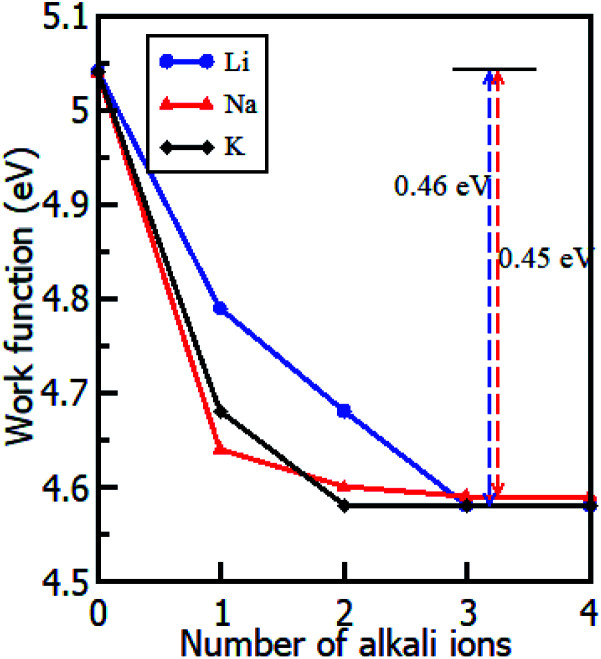
Calculated workfunction of the Gr–HfS_2_ heterostructure as a function of increasing intercalant concentration.

The intercalation of alkali atoms in the Gr–HfS_2_ heterostructure had an influence on the workfunction of the heterostructure, and this is a desirable property for energy storage media. As seen in Fig. 9, the workfunction, calculated using eqn (3), dropped with increasing intercalant concentration up to a constant value of 4.58 eV for both the Li and K intercalant species, and 4.59 eV for Na intercalant. Upon reaching this constant value, the workfunction of the heterostructure had reduced by 460 meV in the case of Li and K intercalation and 450 meV for Na intercalation. This observation is consistent with other studies including Kim *et al.*,^66^ who observed that hole doping in Gr leads to a difference in the workfunction by as much as 400 meV. When the workfunction attains a constant value, it is an indication that there is no more charge imbalance in the system resulting in no further electron flow.

**Fig. 10:**
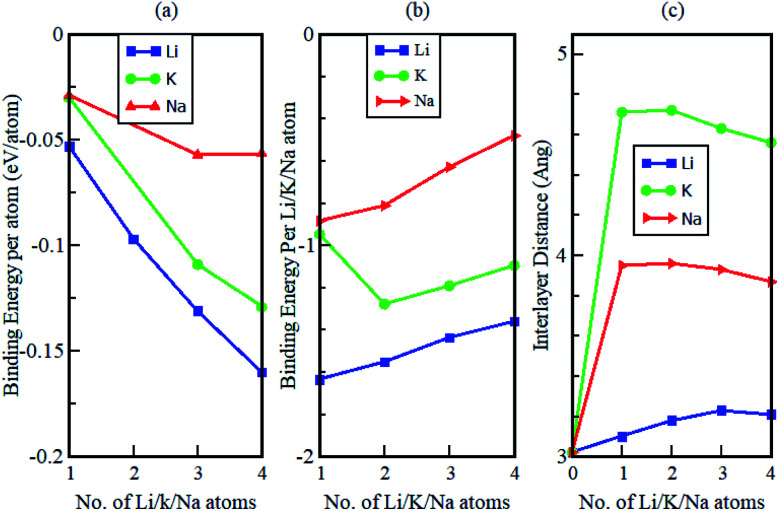
Calculated (a) binding energy per atom, (b) binding energy per intercalant atom and (c) interlayer distance *d*, as a function of K, Na and Li concentration in Gr–HfS_2_ heterostructure.

The study also considered how the binding energy and interlayer distance varied as a function of the number of increasing intercalant atoms (see Fig. 10). The binding energy per atom of the intercalated systems is observed to be highest in Li intercalation and lowest in Na intercalation. Additionally, it was observed that in all instances, the calculated binding energies were negative. This suggests that Li, Na and K intercalation in the Gr–HfS_2_ heterostructure is indeed stable and no phase separation into individual monolayers or the formation of bulk alkali metals is expected. The binding energies per intercalant atom (Fig. 10b), gradually decrease with increasing concentration of the intercalants. This is in line with the behaviour observed in Fig. 9, where an increase in the number of intercalants resulted in a decrease in workfunction. The decrease in binding energy per intercalant atom, can be attributed to the weak electrostatic interaction between the Gr–HfS_2_ host and the intercalant atoms, as a result of enhanced alkali–alkali repulsion as the concentration of intercalants is increased. As the number of adatoms increases, the inter-atomic distances between positively charged ions decreases. For the Li atom the binding energy per Li atom decreases from −1.6 eV to −1.4 eV as the number of intercalated atoms increases from 1 to 4. This can be attributed to the enhanced repulsive interaction between the positively charged Li ions. For K intercalation, the binding energy per K atom initially increases from −0.9 eV to −1.3 eV upon introduction of the first and second K atoms and then decreases. This observation is consistent with an observation made by Demiroglu *et al.*^67^ for K intercalation in Ti_2_CO_2_ Mxene/Gr heterostructure.^67^ For K and Na intercalation, the binding energy per K/Na atoms is initially very low as compared to that of Li. This can be attributed to their ionic radii increasing the interlayer distance between the Gr–HfS_2_ heterostructure layers.

The change in the interlayer distance between the two layers forming the Gr–HfS_2_ heterostructure increases with increasing number of Li ions peaking at 3 Li ions and decreases at 4 Li ions intercalation. For Na and K ions intercalations, the peak was at two ion intercalations (see Fig. 10c). Additionally, the maximum increase in the interlayer separation was found to be 0.21 Å for Li, 0.94 Å for Na and 1.7 Å for K which corresponded to volumetric expansion in the *z*-direction in the order of 6%, 31% and 56.3%, respectively. The 6% volumetric expansion in the case of Li intercalation is comparable with that of graphite anodes which is 10%.^68^ The 31% and 56.3%, for Na and K atoms intercalation is much lower than that for silicon based electrodes which is 280%^69^ or for alloy-type anodes which is 260% for germanium (Ge) and tin (Sn), and 300% for phosphorus (P).^68^ These observations indicate that the Gr–HfS_2_ heterostructure is likely to possess a reversible reaction process in the case of Li, Na and K intercalation, which is another essential property for rechargeable ion batteries. This attribute also implies that Li intercalation in Gr–HfS_2_ heterostructure effectively overcomes the volume expansion problem faced by electrode materials.

### 3.5 Alkali atom diffusion through the heterostructure

**Fig. 11:**
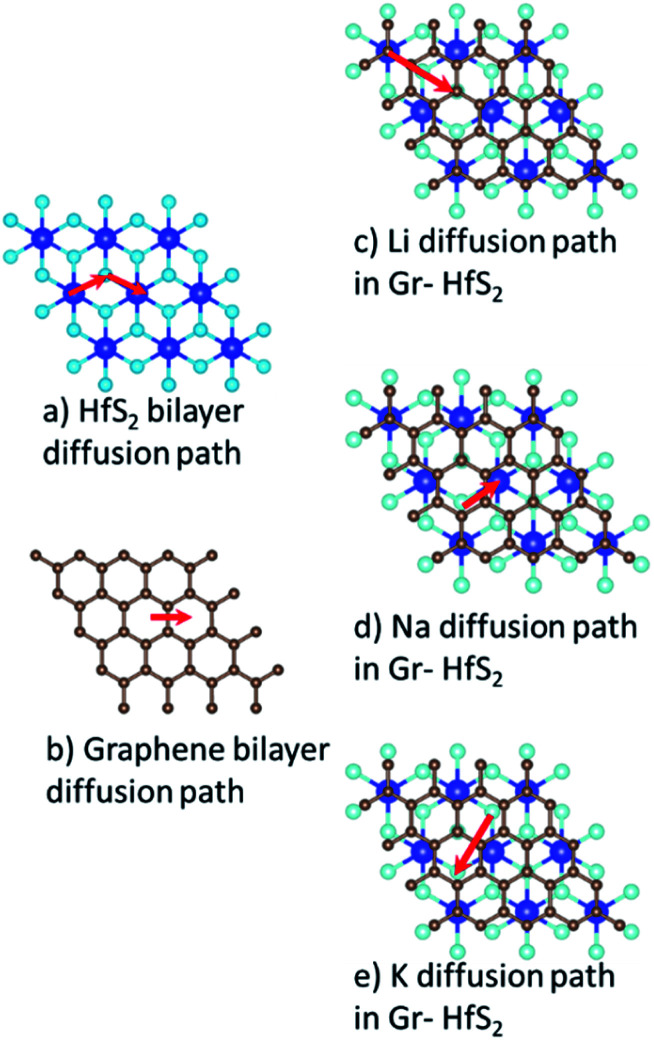
Minimum energy diffusion paths associated with each intercalant species.

The charge/discharge rates of batteries predominantly depend on the ion diffusion in the electrode materials, which further determines the mobility of the adatoms. A smaller energy barrier facilitates faster ionic diffusion and poor diffusivity leads to significant structural damage with continued cycling, which consequently affect the lifetime of the battery.^70^ To investigate the migration/diffusion of the Li, Na and K atoms through the heterostructure, we first located the lowest energy site and then studied the pathways between this site and adjacent sites. Based on the length of the pathways, 3–5 images were employed between various distinct paths as shown in Fig. S2 of the ESI.† The minimum energy path between the two adjacent points gave the energy barrier between them. The energy barriers associated with the intercalants during their migration using different paths are presented in Table S1 (in the ESI).† From Table S1† it is evident that both Li and Na preferred PATH 2 while K preferred PATH 3. Bilayer Gr and HfS_2_ preferred PATH 1. These paths are shown in Fig. 11. From the values of Table S1 (in the ESI),† the minimum diffusion energy barrier associated with the intercalated heterostructure systems for Li, Na and K are, respectively, 0.22 eV, 0.28 eV and 0.05 eV, all these values are lower than for Li ion on graphite (0.42 eV)^71^ and on commercially used anode materials based on TiO_2_ (0.32–0.55) eV.^72^ The lower diffusion energy barriers on the heterostructure systems indicates higher mobility and hence improved battery performance for the heterostructure.

**Fig. 12:**
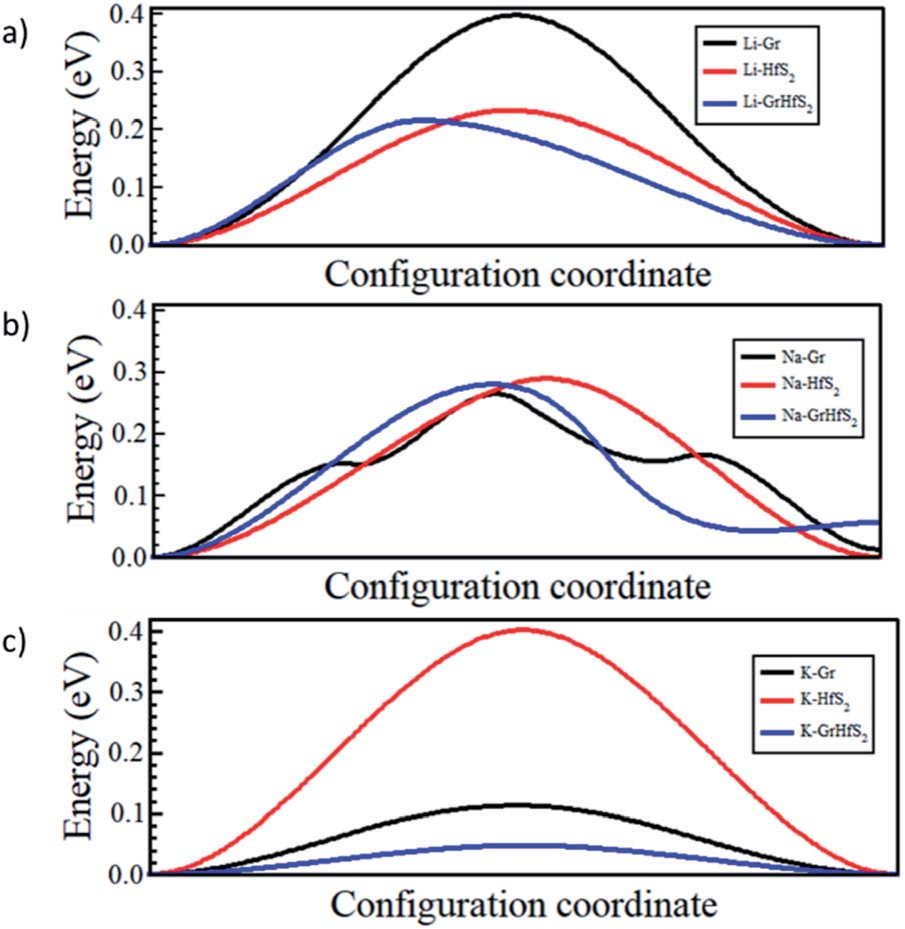
Intercalant diffusion profiles for paths shown in Fig. 11.

Fig. 12 shows the respective diffusion barrier energy profiles associated with respective minimum energy paths in Fig. 11. Of note is that the diffusion energy barriers are lower in the Gr–HfS_2_ heterostructure compared to both bilayer Gr and HfS_2_. It is important to note that the diffusion path for Na through the heterostructure (see Fig. 12b) has a minimum slightly above the minimum of the other paths. This is an indication that the Na adsorbs onto a metastable site and not a global minimum at the end of its migration. The diffusion energy barriers in the heterostructure systems are lower for Na and K ions than Li for the respective minimum energy pathways due to the stronger binding of Li intercalation as seen in Fig. 10. Strong binding energies are expected to pin the atoms on the intercalant site. In order to move the intercalant between sites, a certain amount of energy is required to overcome the adsorption interaction at the site. Hence moving Li, which is the most strongly bonded metal, requires a larger energy threshold to be overcome than the equivalent process for Na and K. The increased interlayer distance in the potassium intercalated heterostructure is also expected to enhance the diffusion process, leading to the potassium intercalated heterostructure having the lowest energy barrier.

### 3.6 Electrochemical properties

**Fig. 13:**
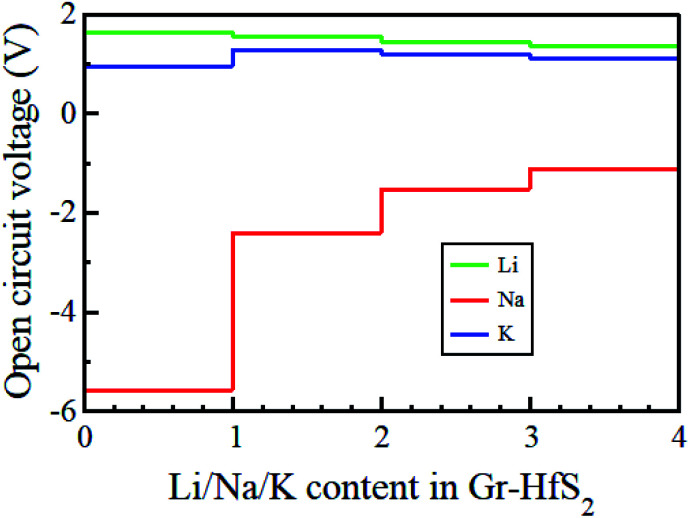
Open circuit voltage profiles of Li, Na, and K intercalation in Gr–HfS_2_ heterostructures as a function of alkali atom concentration.

In order to gain insights into the electrochemical properties of the Li, Na and K intercalation process into the Gr–HfS_2_ heterostructure, the open-circuit-voltage (OCV) was determined. The OCV value gives a measure of the performance of a battery, and was calculated from the energy difference based on the equation:5
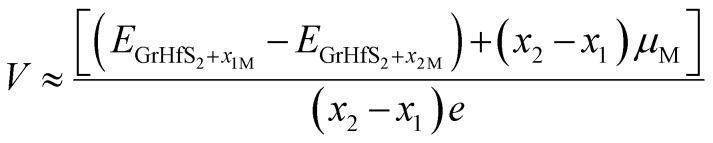
where *E*_GrHfS_2_+*x*_1_M_ and *E*_GrHfS_2_+*x*_2_M_ are the total energies of the Gr–HfS_2_ heterostructure with *x*_1_ and *x*_2_ alkali atom intercalated, respectively, *μ*_M_ is the chemical potential of Li/Na/K atom and *e* denotes the elementary charge quantity.^73–75^ The chemical potential of Li/Na/K atom is approximately equal to the total energy per Li/Na/K atom, and hence this was the value used in eqn (5).^73,75^ The chemical potential of Li/Na/K atom is approximately equal to the total energy per Li/Na/K atom, and hence this was the value used in eqn (5).^73,75^ The calculated voltage profiles of the three considered systems are shown in Fig. 13. It is observed that the voltage decreases gradually from 1.64 V to 1.36 V as the number of Li adatoms increases, while that of K intercalated system initially increase from 0.94 V to 1.28 V then decreases to 1.10 V. The calculated average voltage profile is 1.49 V for Li and 1.13 V for K intercalated systems. The voltage is positive for all Li and K concentrations, indicating that the Li and K intercalated system can be fully intercalated. The voltage associated with Na intercalation into Gr–HfS_2_ heterostructure is negative, an indication that Na intercalation is chemically unstable in the Gr–HfS_2_ heterostructure. The calculated voltage values for all systems correlate with the binding energy values per atom, presented in Fig. 10a. The highest voltage is found for Li as this system has the largest binding energy, (see Fig. 10a). The lowest voltage is found for Na as this system has the least binding energy per atom, (see Fig. 10a). Our results indicate that Li and K intercalation in Gr–HfS_2_ heterostructure can be exploited in low voltage applications.

### 3.7 Charge density distribution and population analysis

**Fig. 14:**
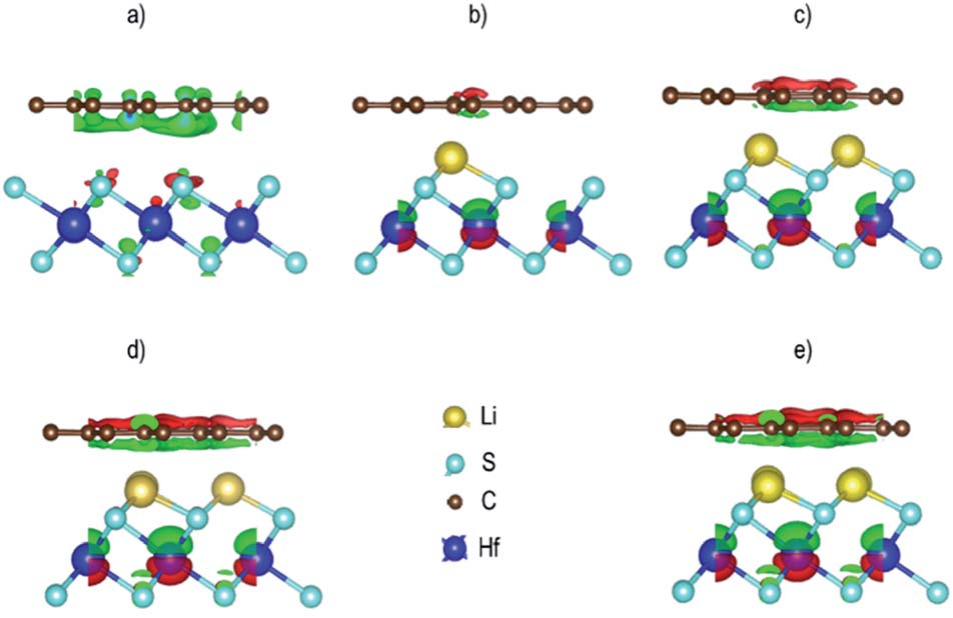
Charge density difference for the Gr–HfS_2_ heterostructure systems (a) without intercalant, with (b) one, (c) two, (d) three and (e) four Li intercalants. The green isosurface indicates charge accumulation while the red isosurface indicates charge depletion at 0.004 eÅ^−3^ for all the intercalated systems and 0.4 eÅ^−3^ for the pristine system.

In order to understand the mechanism of the charge distribution and charge transfer between Gr and HfS_2_ monolayers in the Gr–HfS_2_ heterostructures, we calculated the charge densities difference (Δ*ρ*) using the relation (eqn (6)):

6 Δ*ρ* = Δ*ρ*_Gr/HfS_2__− Δ*ρ*_Gr_ − Δ*ρ*_HfS_2__

where Δ*ρ*_Gr/HfS_2__ is the charge density of the heterostructure, Δ*ρ*_Gr_ is the charge density of Gr and Δ*ρ*_HfS_2__ is the charge density of Hafnium disulfide. The resulting charge density difference distribution is shown in Fig. 14. Evidence of charge distribution between the two layers is observed with and without the Li intercalants. Charge accumulation is represented by the green iso-surface while charge depletion is represented by the red iso-surface. It is worth noting that the iso-surface level for the pristine Gr–HfS_2_ heterostructure was 0.0004 eÅ^−3^, while those of the rest was 0.4 eÅ^−3^. The iso-surface between Gr and HfS_2_ layer is a charge accumulation region. As the number of intercalants increase, (as we move from Fig. 14b to d) the amount of charge accumulated increases while the regions with depletion around Hf ions also increases, this can be attributed to the reduction in the workfunction of the heterostructure as the number of intercalants is increased from one to four (see Fig. 9). A reduction in the work-function makes it easier for charge to be lost to the surface, hence the increase in the charge depletion and charge accumulation regions as the number of ions increases. Within the HfS_2_ layer the charge accumulation mainly occurs around the sulphur atoms, an indication that these atoms gain charges. A similar observation has been made for the Gr/MoS_2_ ^76^ and tungsten sulfide (Ws_2_)/Gr,^77^ heterostructures. It has also been shown that tungsten diselenide (WSe_2_) is a weak acceptor of electrons upon contact with Gr, in a WSe_2_/Gr heterostructure.^78^

In order to get further insight into the charge transfer between the heterostructure and the intercalated adatoms, a Lowdin population analysis was performed, and the results obtained are shown in Tables 4 and 5. It was observed that upon Li/K intercalation, charge was transferred to Li/K adatom from both the Graphene and HfS_2_ layers leaving them negatively charged. In both cases, the charge was mainly donated by the Gr layer than the HfS_2_ layer. The amount of charge transferred gradually reduced as the number of intercalants increased. This could be attributed to the increased repulsion due to decreased distance between ions as their number is increased. This in turn reduces the interaction between the ions and the host material.

**Table 4:** Lowdin charge difference associated with varying levels of Li intercalation

	Charge difference			
	1 Li intercalants	2 Li intercalants	3 Li intercalants	4 Li intercalants
HfS_2_	−0.273	−0.277	−0.310	−0.313
Gr	−0.895	−0.886	−0.876	−0.779
Li	3.714	3.512	3.393	0.784

**Table 5:** Lowdin charge difference associated with varying levels of K intercalation

	Charge difference			
	1 K intercalants	2 K intercalants	3 K intercalants	4 K intercalants
HfS_2_	−0.248	−0.281	−0.303	−0.304
C	−0.883	−0.874	−0.869	−0.767
k	9.486	9.369	9.289	8.719

## 4 Conclusions

This study has systematically investigated the prospects of Gr–HfS_2_ heterostructure, as an electrode material for alkali ion (Li, Na and K) batteries, using first-principles calculations with vdW-DF2 corrections. The stability of the heterostructure upon alkali ion intercalation is confirmed by the negative binding energy values for all the intercalated atoms and also by donation of a significant amount of charge, as confirmed by both the charge density difference and Lowdin population analysis. The volumetric expansion due to the intercalant species was found to be 6%, 31% and 56.3%, for Li, Na and K, respectively, suggesting that the Gr–HfS_2_ heterostructure possess a reversible reaction ability. Diffusion energy barriers confirm the advantage of Gr–HfS_2_ heterostructure over graphene and HfS_2_ bilayer systems. Relatively low diffusion energy barriers ranging between 0.22–0.39 eV for Li, 0.05–0.09 eV for K and 0.28–0.74 eV for Na were determined for the intercalated Gr–HfS_2_ heterostructure. This implies high charge/discharge rate in battery applications. Li intercalation in Gr–HfS_2_ is attractive for rechargeable ion battery applications as it overcomes the volume expansion problem faced by many electrode materials. The findings of this study suggest that it is possible to develop next-generation anode materials with ultrafast charging/discharging rates using Gr–TMDC heterostructure.

## Conflicts of interest

There are no conflicts to declare.

## Supplementary Material

RA-010-D0RA04725B-s001
