# Supplementary material for: Two-dimensional graphene–HfS2 van der Waals heterostructure as electrode material for alkali-ion batteries
Source: RSC Adv. 2020 Aug 17;10(50):30127–38. doi: 10.1039/d0ra04725b (PMC9056275; doi:10.1039/d0ra04725b)
Supplement: RA-010-D0RA04725B-s001 [file RA-010-D0RA04725B-s001.pdf]

Electronic Supplementary Material (ESI) for RSC Advances.
This journal is © The Royal Society of Chemistry 2020

Supplementary Information

Two-Dimensional graphene-HfS_2 van der Waals heterostructure as electrode material for Alkali-ion batteries.

Gladys King'ori[a, b], Cecil N M Ouma[c], Abhishek K Mishra[d], George O Amolo[b], Nicholas W Makau[a].

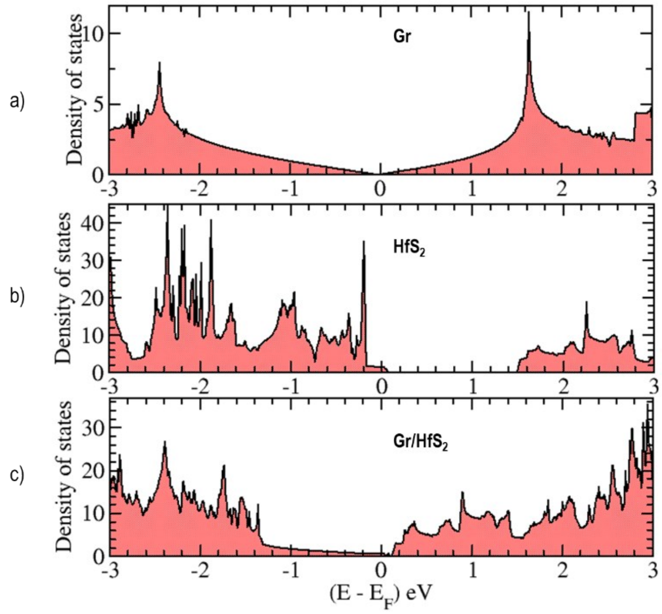

Figure S1 Calculated density of states of (a) 3×3×1 Gr supercell, (b) 2×2×1 HfS_2 supercell and (c) Gr-HfS_2 heterostructure system.

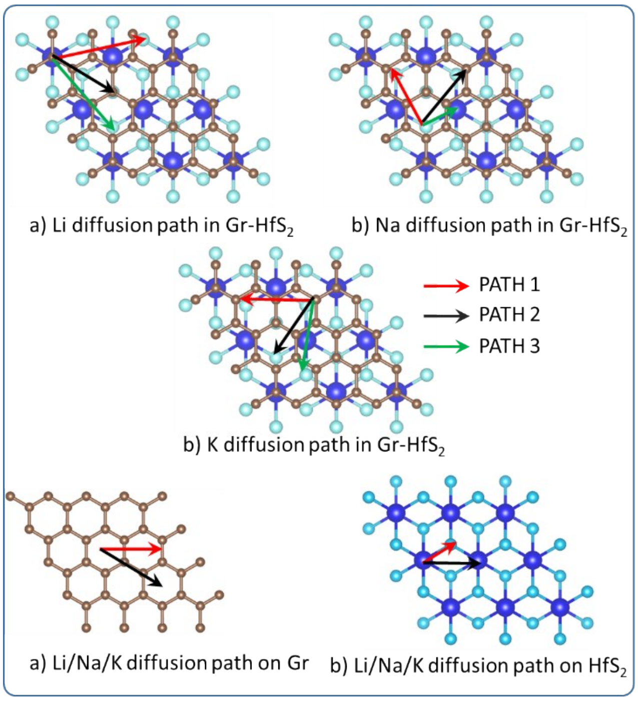

Figure S2: Migration pathways of a) Li b) Na and c) K diffusion between the Gr-HfS_2 heterostructure layers. d) and e) are the migration pathways for Li, Na and K diffusion between the bilayers of Gr HfS_2 respectively.

Table S1: Energy barriers (in eV), associated with diffusing adatoms through the various paths shown in Figure S2.

	PATH 1	PATH 2	PATH 3
Li diffusion through $GrHfS_2$	0.39	**0.22**	0.26
Na diffusion through $GrHfS_2$	0.46	0.74	**0.28**
K diffusion through $GrHfS_2$	0.06	**0.05**	0.09
Li diffusion through Gr bilayer	**0.39**	0.42	
Na diffusion through Gr bilayer	**0.26**	0.54	
K diffusion through Gr bilayer	**0.11**	0.13	
Li diffusion through HfS_2 bilayer	**0.10**	0.69	
Na diffusion through HfS_2 bilayer	**0.32**	0.50	
K diffusion through HfS_2 bilayer	**0.19**	0.40	
